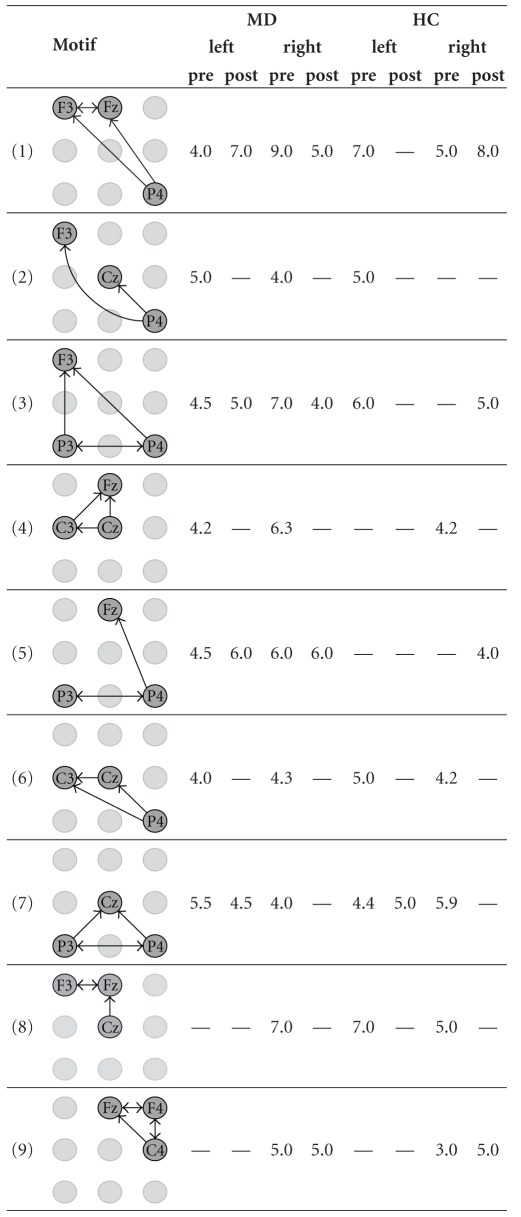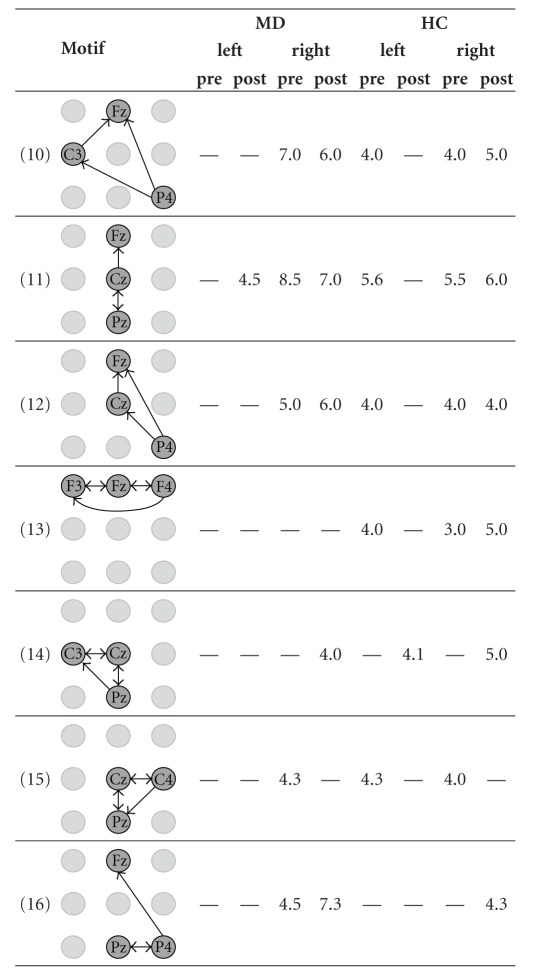# An Analytical Approach to Network Motif Detection in Samples of Networks with Pairwise Different Vertex Labels

**DOI:** 10.1155/2012/910380

**Published:** 2012-05-14

**Authors:** Christoph Schmidt, Thomas Weiss, Christian Komusiewicz, Herbert Witte, Lutz Leistritz

**Affiliations:** ^1^Institute of Medical Statistics, Computer Sciences and Documentation, Jena University Hospital, Friedrich Schiller University Jena, Bachstrasse 18, 07743 Jena, Germany; ^2^Department of Biological and Clinical Psychology, Friedrich Schiller University Jena, Am Steiger 3, Haus 1, 07743 Jena, Germany; ^3^Institut für Softwaretechnik und Theoretische Informatik, TU Berlin, Fakultät IV, Elektrotechnik und Informatik, Sekr. FR 6-1, Franklinstrasse 28/29, 10587 Berlin, Germany

## Abstract

Network motifs, overrepresented small local connection patterns, are assumed to act as functional meaningful building blocks of a network and, therefore, received considerable attention for being useful for understanding design principles and functioning of networks. We present an extension of the original approach to network motif detection in single, directed networks without vertex labeling to the case of a sample of directed networks with pairwise different vertex labels. A characteristic feature of this approach to network motif detection is that subnetwork counts are derived from the whole sample and the statistical tests are adjusted accordingly to assign significance to the counts. The associated computations are efficient since no simulations of random networks are involved. The motifs obtained by this approach also comprise the vertex labeling and its associated information and are characteristic of the sample. Finally, we apply this approach to describe the intricate topology of a sample of vertex-labeled networks which originate from a previous EEG study, where the processing of painful intracutaneous electrical stimuli and directed interactions within the neuromatrix of pain in patients with major depression and healthy controls was investigated. We demonstrate that the presented approach yields characteristic patterns of directed interactions while preserving their important topological information and omitting less relevant interactions.

## 1. Introduction

Many processes and systems have a network structure that consists of interacting units which can be represented as a graph. Accordingly, analysis from a graph theory perspective has recently become a focus of research as unique insights are obtained into the working and organization of various complex systems. For example, in the study of cellular signaling pathways associated with cancer it was revealed that the activity of p53, a central tumor suppressor that regulates many different genes, can only be understood by considering associated tangled signaling networks in their entirety and the position of p53 integration within these networks, instead of considering interactions of p53 with single network components [1]. In synthetic biology, “network thinking” is crucial for the understanding and assembly of biological modules that are used in engineering cellular machines to perform tasks such as producing drugs or acting as biosensors that detect toxic compounds [2, 3]. In epidemiology, considerable effort has been directed to examining mechanisms by which the topology of networks of contacts between individuals affects the spreading of diseases in order to find ways to predict and control the propagation of infections [4]. The progress made in modern network theory has also led to new applications in the neurosciences that attempt to find explanations for previously inadequately understood higher level brain processes [5]. Topological properties of anatomical and functional connectivity networks have been studied to obtain understanding on the organization of cortical areas. It has been suggested that brain systems exhibit a small-world topology which implicates simultaneous and well-balanced segregation and integration of information processing and results in minimization of wiring costs for economical brain performance [6, 7]. Disturbances of this evolutionary optimized topology of cortical networks have been reported to alter functional connectivity and thereby cause neuropsychiatric disorders such as Alzheimer's disease [8], schizophrenia [9, 10], or epilepsy [11] that are often described as disconnection syndromes [12–14]. Pathological abnormalities in cortical network organization may be quantified by network measures which might act as useful diagnostic markers. An overview of measures that quantify global and local network topology can be found for example in [15–17].

Network motifs, the subject of this publication, constitute an exceptional influential measure of local network topology that enables a detailed description of overrepresented local patterns of interconnections [18, 19]. Subsequently, these overrepresented subnetworks may be linked with a potential functional contribution to the global functionality of the entire network. The functionality of a system is to some extent enclosed or encoded in the topology of its representing network; as a consequence, it is assumed that individual networks (or at least networks of a certain type) possess characteristic combinations of recurring small, connected subnetworks that act as functional meaningful building blocks or as elementary computational circuits for information processing [18, 20, 21]. The importance of the functional contribution of a subnetwork is assumed to be reflected in an overrepresented, nonrandom and perhaps conserved occurrence of it in its network. According to this, a functional constraint for subnetworks correlating with their nonrandom appearance is robustness to small perturbations in order to enable robust network performance, especially in biological networks [22]. Network motif detection has been directly adopted into a variety of different research fields; interesting results have been obtained by its application to study structure-function relationships and design principles in networks from various domains such as protein-protein interaction networks, the World Wide Web, electronic circuits, synaptic neuronal networks, and transcriptional gene regulation networks [18–20, 23, 24]. A slightly modified variant of motif detection was used to investigate structural motifs and the instances of functional motifs contained within them in the context of anatomic brain networks of macaque visual cortex, macaque cortex, and cat cortex [25].

The original network motif detection approach attempts to find significant frequent subnetworks in one single-directed network with (usually many) unlabeled vertices that are indistinguishable from each other. Original network motif detection basically consists of three computationally expensive subtasks.

1. Exhaustively enumerating [18] or sampling (estimating) [26, 27] the number of occurrences of each subnetwork of size *k* (a subnetwork induced by a vertex set of *k* vertices) in the input network. This quantity is affected by the kind of vertex and edge overlap one allows for counting different matches of a subnetwork [28, 29]. Typically, one allows for nonidentical counting (arbitrary overlaps) of subnetworks where the downward closure property does not hold. This dramatically increases the number of subnetwork occurrences in a network compared to counting only edge-disjoint subnetworks, that is, subnetworks that do not share any edges. Therefore, even in comparably small networks, the number of subnetwork occurrences is potentially large due to its exponential increase with the size of the input network. Moreover, the number of *k*-subnetworks in a network grows very fast with *k*. In order to avoid impractical running times and difficulties with assessing functional roles of larger subnetworks, the size parameter *k* for subnetworks is usually chosen to be 3 or 4.
2. The second subtask in network motif detection encompasses determining graph isomorphism for grouping found subnetworks into equivalence classes. It is believed that graph isomorphism cannot be solved in polynomial time. Several algorithms for solving graph isomorphism with miscellaneous performance in practice have been presented [30].
3. The last subtask is assessing statistical significance of subnetwork occurrences. Subnetworks that occur in significantly large numbers in the input network as compared to their occurrence in a large set of null model random networks are accepted to be motifs. The comparison of a network with a set of associated random networks should reveal deviations of network properties such as the number of subnetwork occurrences from randomness. Therefore, the underlying random graph model has to be chosen carefully, because it is this model that specifies the notion of randomness. Hence, it has to strike a balance between preserving functional constraints and characteristics of the input network while at the same time comprising random edge patterns so that at best no subnetwork appearance is being favored [31]. The commonly employed random graph model preserves the incoming and outgoing degree sequence—an important characteristic of single vertices—of the input network and the associated random networks are usually generated either by the configuration model (stubs matching) algorithm [32–35] or by an Markov chain Monte Carlo edge rewiring (switching) algorithm [18, 32, 33, 36, 37]. A subnetwork occurrence is defined to be significant if it occurs a certain multiple of standard deviations more often in the input network than would be expected in the set of random networks. This is expressed by the *z*-score which relates the count of a subnetwork in the input network to the mean and the standard deviation of its count in the set of random networks [25, 26, 28]. Making use of *z*-scores for assigning statistical significance to subnetwork occurrences is flawed by the unsafe assumption being made that subnetwork occurrences follow a normal distribution [28] and it was shown that this is not always the case [38]. Statistical significance of subnetwork occurrences might also be assessed by computing whether the probability that a subnetwork occurs an equal or greater number of times in a random network than in the input network is lower than a cutoff value [18, 19].

We present a novel approach to network motif detection that differs from the original approach and avoids some of its limitations. In this approach, we intend to detect subnetworks that are motifs for a sample of directed networks where each network possesses the same pairwise different vertex labels. Furthermore, we intend to not discard these vertex labels but rather preserve the functional important topological information associated with them. Topological information has already been used in previous studies to visualize spatio-temporal connectivity structures [39–41]. Another advantage of this approach is to analytically compute the statistical significance of subnetwork counts, which would save considerable computation time since no random network ensembles have to be generated and no subnetwork counts have to be obtained from them. Motifs that are obtained by this analysis would yield a description of locatable and characteristic interaction patterns of a sample of networks and moreover could be used as a distinguishing characteristic to reveal sample-specific differences in network topology. We demonstrate that this approach may be applied to investigate networks that model pain processing in a group of patients with major depression (MD) and a group of healthy controls (HCs) in order to acquire deeper insight into the intertwined relationship between pain and depression where many details are poorly understood. It is known that chronic pain and major depression are correlated since depression is a common comorbidity of chronic pain and often chronic pain is an additional symptom of depressed patients [42, 43]. It has been confirmed by some studies that thresholds for acute painful stimulation are lower in depressed patients than in healthy controls [44, 45], whereas other studies found the opposite, namely, increased thresholds in depressed patients [42, 46–49]. The physiological basis for pain perception, pain processing, and the sensitivity to painful stimuli of depressed patients remains unclear. It is hypothesized that in depressed patients the processing of painful stimuli in the so-called neuromatrix of pain [50] and consequently the effective connectivity might be altered [47].

We denote the networks from the samples we investigate in the present study by effective connectivity networks (ECNs). The network data originates from a previous study in which we used effective connectivity analysis to investigate the processing of moderately painful intracutaneous electrical stimuli and directed interactions within the neuromatrix of pain in both groups, MD and HCs, by means of frequency selective generalized partial directed coherence (gPDC) [51]. The intricacy of the connectivity patterns in ECNs does not allow for immediate interpretation. In order to overcome the inability to qualitatively describe the intricate wiring patterns found in ECNs and to shed light on elementary directed interactions in both groups we use our novel approach as a filter that detects labeled network motifs and omits less important interactions. We demonstrate that in this way we gain interesting new insights into the relationship between chronic pain and depression, which is currently inadequately understood.

## 2. Materials and Methods

 The present study directly follows up on the EEG experiments and the connectivity analysis published in [51] and is based on the same materials. For the sake of completeness, a short sketch of the baseline characteristics is given in this section.

### 2.1. Subjects

 Eighteen patients (10 women, 8 men) with major depression (mean age ± standard deviation: 38.9 ± 15.5 years) and 18 sex- and age-matched healthy control subjects (39.3 ± 14.8 years) participated in this study. Patients were treated in a specialized psychiatric ward for mood disorders. Major depression was established by a staff psychiatrist according to DSM IV criteria using a structured interview, and the Beck depression inventory (BDI) was also administered. BDI scores of patients ranged from 19 to 48 (29.4 ± 9.7); scores of control subjects were all below five (2.1 ± 1.5). All subjects were right handed. Nine patients were treated with antidepressive medication (5 patients received selective serotonin reuptake inhibitors SSRI; 4 patients norepinephrine and serotonin reuptake inhibitors NaSRI) while the remaining participants did not receive any medication. One patient and two controls had to be excluded from the experiments because they could not follow the test protocol.

### 2.2. Connectivity Analysis

 All subjects were electrically stimulated intracutaneously at the tip of the middle fingers of both the right and the left hand. The intensity level was adjusted between 10 *μ*A and 1 *μ*A. Stimuli consisted of a bipolar rectangular pulse of 10 ms duration. Participants were requested to rate each electrical stimulus on a scale ranging from 0 to 6 (0 = no sensation; 1 = just perceived, not painful; 2 = clearly perceived, but not painful; 3 = low pain; 4 = moderate pain; 5 = strong pain, but tolerable; 6 = unbearable pain) [52, 53]. The pain threshold was defined as the intensity yielding a sensation described as a sharp painful pinprick, corresponding to a rating of “3.” The EEG was recorded continuously during the electrical stimulation from 60 electrodes, referenced to Cz, using a standard EEG cap (Easy Cap, Falk Minow Services, Germany) based on an extended International 10–20 system. Finally, nine electrodes F3, Fz, F4, C3, Cz, C4, P3, Pz, and P4 (re-referenced to linked ears) that are situated above some of the important regions of pain processing, attention, and depression (frontal, central, and parietal brain regions) were used. Eye movement and muscle activity artifact contaminated single trial somatosensory evoked potentials (SEPs) were excluded, which resulted in an exclusion of three data sets since there were not enough artifact-free trials left for a reliable connectivity analysis. In order to compare the pre- and post-stimulus condition, signal sections of 700 ms duration were extracted pre- (700 ms before onset to the onset of stimulus, i.e., −700 ms to 0 ms) as well as post-stimulus onset (from stimulus onset to 700 ms after stimulus onset, i.e., 0 ms to 700 ms). These signal sections provided the data basis for the connectivity analysis. To assess the effective connectivity between each directed pair of the nine electrodes, the generalized partial directed coherence (gPDC) [54] was applied. The frequency range of interest for the SEP analysis was determined to be in the delta-, theta- and the alpha-bands (1 to 13 Hz) since the signal power is mainly situated in this frequency range. For a consolidated analysis, the gPDCs of the corresponding frequencies were pooled to one quantity by averaging with respect to the frequency range of interest. Thus, for each of the 72 possible directed interactions, one gPDC value results each. Finally, the effective connectivity we are interested in is given by significantly increased gPDC values. A detailed description of the entire procedure may be found in [51].

### 2.3. Effective Connectivity Networks

 In this study, we refine the view of this effective connectivity data by examining effective connectivity from a different perspective: we model each participant's directed interactions, which are given by significant gPDC values, as effective connectivity networks (ECNs). The topology of ECNs consequently represents a valuable source of information about the relationship between pain and depression, which is incompletely understood. Subsequently, we apply our network motif detection approach to group-specific samples of ECNs to find patterns of directed interactions that may be considered as a characteristic of the group of patients or the group of controls, respectively. These characteristic patterns may shed light on the basic neural activity which occurs during the processing of painful stimuli in patients with major depression and in the healthy controls.

Due to the nature of the underlying EEG experiment, eight samples of ECNs may be considered. They are defined by all combinations of the group assignment (MD—patients suffering from major depression versus HC—healthy control subjects), the stimulated side (left versus right), as well as the time window with respect to the stimulus conditions (pre—time window before noxious stimulation versus post—time window directly following the stimulation, i.e., including the processing of the noxious stimulus). The nomenclature is MD-pre-left, MD-pre-right, MD-post-left, MD-post-right, HC-pre-left, and so forth. The sample size for the MD-post-right sample is fifteen, where the sample size equals sixteen for all other samples.

**Figure 1:**
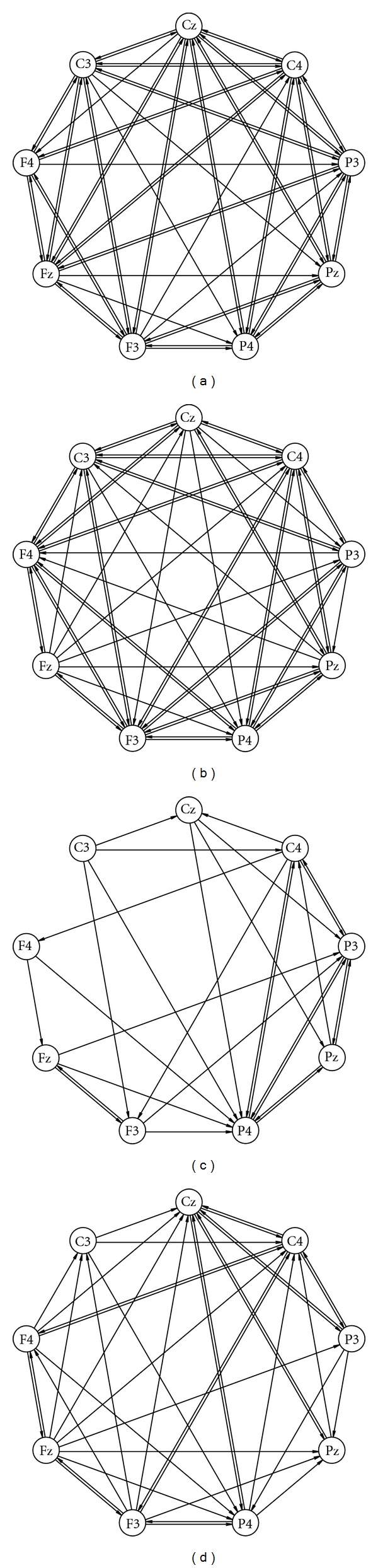
Examples of effective connectivity networks (ECNs). ECNs consist of nine vertices corresponding to EEG-electrodes that are pairwise differently labeled by associated EEG-electrode identifiers and directed edges corresponding to directed interactions between EEG-electrodes as indicated by significant gPDC values. In their wiring patterns, samples of ECNs contain intrinsic information about the processing of painful electrical stimuli in a group of patients suffering from major depression and a group of healthy controls.

As directed graphs, effective connectivity networks consist of a nonempty finite set *𝒱* of vertices and a finite set *ℰ* of ordered pairs of distinct vertices called arcs or edges. An ordered pair (*u*_*i*_, *u*_*j*_) is called directed edge if it leaves vertex *u*_*i*_ and enters vertex *u*_*j*_. It is denoted by *u*_*i*_ → *u*_*j*_ and *u*_*i*_ is called the tail and *u*_*j*_ is called the head of the edge. An ECN is represented by its adjacency matrix *𝒜* of size 9 × 9 where *𝒜*_*ij*_ = 1 if and only if the ECN contains the directed edge *u*_*i*_ → *u*_*j*_. Accordingly, a mutual edge is indicated by two entries in the adjacency matrix *𝒜*_*ij*_ = 1 and *𝒜*_*ji*_ = 1 and is denoted by *u*_*i*_↔*u*_*j*_. The effective connectivity networks are built by abstracting EEG-electrodes as vertices and modeling associated directed interactions by directed edges between those vertices. The ECNs of the present study are small networks, each consisting of the same set of nine vertices that are pairwise differently labeled by associated EEG-electrode identifiers. For our approach, it is crucial that, due to the vertex labeling, all vertices are different. Most ECNs exhibit dense and intricate patterns of directed interactions. The mean number of edges in an ECN is 36.79 out of 72 possible edges. Moreover, ECNs do not contain multiple edges (edges with the same tail and the same head) and loops (edges whose tail and head coincide). Due to the properties of ECNs, their adjacency matrices are asymmetrical with 0 entries on the main diagonal. With two exceptions, all ECNs are connected networks. Examples of ECNs are depicted in Figure 1.

### 2.4. Network Motif Detection in a Sample of Directed Networks with Pairwise Different Vertex Labels

Dealing with network samples of directed networks with identical pairwise different vertex labeling instead of single networks without such labeling imposes certain constraints on the approach to network motif detection and also on the definition of a network motif. The most important constraint is that each subnetwork can occur at most only once in a single network, which affects the statistical analysis of subnetwork occurrences. It is not possible to assign significance to subnetwork counts in one network or in very small samples of networks. Therefore, motif detection in a sufficiently large sample of networks constitutes not only a novel approach to reveal common topological characteristics of all sample elements but is also a necessity. Given the pairwise different vertex labeling, two subnetworks are identical if and only if they share the same set of edges, that is, they have identical adjacency matrices. Therefore, isomorphic subnetworks do not exist and consequently it is unnecessary to address the problem of determining graph isomorphism for subnetworks. It is completely different for networks without vertex labeling. In the unlabeled case, different topological equivalence classes of subnetworks exist, also called motif classes or identities and each of them might consist of isomorphic subnetworks. For example, there are 13 equivalence classes of 3-subnetworks without vertex labeling comprising a total of 54 isomorphic subnetworks [18, 25]. In contrast, if the 3-subnetworks had pairwise different vertex labels, there are 54 different such subnetworks, each corresponding to one of the isomorphic subnetworks of the unlabeled case.

In order to keep the constraints given by the vertex labeling, one has to extend the original notion of network motifs [18, 19] to define the special case of network motifs of a sample of directed networks with pairwise different vertex labeling. Therefore, we define network motifs as small connected subnetworks which differ in their set of edges, as opposed to differing in their patterns of interconnections only, which appear in their sample of networks significantly more often than in random networks according to a suitable random graph model. In this way, we take the vertex labeling into account that does not only give us an advantage with respect to the computational complexity of our task to detect motifs but also has the important advantage of conserving the positional information of motifs in the network. This positional information is somehow associated with underlying neural processes and, therefore, is important for a subsequent functional interpretation of the results. If vertex labeling is discarded, then the only information obtained from motifs is about significant patterns of directed influences between EEG-electrodes. However, their localization would be missing which makes it unfeasible to functionally compare different instances of a motif in sets of vertex labeled networks.

#### 2.4.1. Exhaustive Enumeration of Subnetworks

Let *𝒩* = (*𝒩*_1_,…, *𝒩*_*n*_) be a sample of vertex-labeled directed networks *𝒩*_*i*_ = (*𝒱*, *ℰ*_*i*_) all having the same set *𝒱* of *ν* vertices and a particular set *ℰ*_*i*_ of directed edges. *𝒜* ^*i*^ denotes the adjacency matrix that represents network *𝒩*_*i*_. The first step in our approach is to explicitly enumerate all subnetworks of a certain size *ν*_*S*_ ≥ 2 in every network *𝒩*_*i*_ which is feasible due to the size of the networks and the sample size. Thereby, for each member network *𝒩*_*i*_, every combination of *ν*_*S*_ vertices is investigated with respect to the subnetwork induced by it. Subsequently, the number of occurrences of each induced subnetwork over the entire sample is counted. Based on these subnetwork counts, we analytically assign significance to subnetworks.

#### 2.4.2. Testing Significant Subnetworks Occurrences

 In order to identify subnetworks that occur significantly more often than expected in random networks, a suitable model for such random networks is required. Such a model is called null model. Due to pairwise different vertex labels, each subnetwork can occur at most once in a network. Thus, the usual *z*-score approach [25, 26] cannot be applied. However, a suitable null model for labeled networks may be derived, if a sufficiently large sample of networks is available.

Let 0 ≤ *k*_*i*_ ≤ *ν*(*ν* − 1) be the number of edges of *𝒩*_*i*_, and let

(1) $$\begin{array}{c} q=\frac{1}{n\nu \left(\nu -1\right)}\overset{n}{\underset{i=1}{\sum }}k_{i} \end{array}$$

be the normalized mean number of edges of the sample *𝒩*. Then, the i.i.d. variables *𝒜*_*kl*_^0^, 1 ≤ *k* ≠ *l* ≤ *ν*, with

(2) $$\begin{array}{cc} P\left({𝒜}_{kl}^{0}=1\right) & =q,\\ P\left({𝒜}_{kl}^{0}=0\right) & =1-q, \end{array}$$

describe a random network *𝒩* ^0^ = (*𝒱*, *ℰ* ^0^) with a mean number of edges *qν*(*ν* − 1). It provides the basis of the null model. Let *S* be an arbitrary subnetwork with at least *ν*_*S*_ ≥ 2 vertices of the set *𝒱* and *η*_*S*_ edges. Obviously, the subnetwork *S* can exhibit at most *η*_*S*_max⁡__ = *ν*_*S*_(*ν*_*S*_ − 1) edges. We are interested in the count that *S* occurs in the sample *𝒩* as subnetwork. For it, we define *n* i.i.d. random variables *X*_*i*_ by

(3) Xi={1,  if  S  is  a  subnetwork  of  𝒩00,  if  S  is  not  a  subnetwork  of  𝒩0.

Assuming the null model, the probability that *S* occurs as a subnetwork of *𝒩* ^0^ is by definition equal to

(4) $$\begin{array}{c} P\left(X_{i}=1\right)=q^{\eta_{S}}\cdot {\left(1-q\right)}^{\eta_{S_{\max}}-\eta_{S}} \end{array}$$

for all *i* = 1,…, *n*. Since all sample networks *𝒩*_*i*_ are associated with the same null model, the count that *S* occurs in the sample *𝒩* as subnetwork is binomially distributed under the null model.

(5) $$\begin{array}{c} \overset{n}{\underset{i=1}{\sum }}X_{i}\sim B\left(n,q^{\eta_{S}}\cdot {\left(1-q\right)}^{\eta_{S_{\max}}-\eta_{S}}\right). \end{array}$$

Finally, all subnetworks of a certain size *ν*_*S*_ are tested with respect to a significant overrepresentation in the sample. Thus, an alpha-adjustment has to be applied. In the present study, generally the Bonferroni-Holm correction [55] with a multiple significance level of *α* = 0.05 was adopted for all multiple test procedures to conservatively control the familywise error rate for all hypotheses at *α* in the strong sense instead of controlling the expected proportion of incorrectly rejected null hypotheses (false discovery rate).

## 3. Results and Discussion

 We applied our approach to detect network motifs in eight group-specific samples of ECNs that were obtained from our effective connectivity data [51]. As a result of dismissing interactions that are by definition less important, we reduce the information of the intricate patterns of directed interconnections of a sample of ECNs. We interpreted network motifs as patterns of characteristic interactions in a sample of ECNs. Because of the spatial information associated with the vertex labels, it makes sense to look even for 2-motifs in order to find significant interactions between two areas covered by the EEG-scheme. Furthermore, we were interested in characteristic interaction patterns that are represented by 3-motifs. We did not aim to detect motifs of a larger size because physiological interpretation of 2-motifs and 3-motifs is already difficult. Hence, detecting larger motifs does not seem to contribute much to the qualitative knowledge about effective connectivity networks. However, from a theoretical point of view, the detection of larger motifs is straightforward given that sufficiently large samples are available. Due to their small number, all 2-motifs detected by our approach could be presented in Table 1. In contrast, due to their large number, only those interesting 3-motifs whose occurrence is sample-specific or which occur in most samples of ECNs are presented in Table 2.

Table 1 illustrates that some 2-motifs represent functional connections that are present in the pre- as well as in the post-stimulus period, both for MD and HC. Typical examples are P4 → C3 or Fz *↔* F4. Such connections might represent parts of the background activity or attentional processes which are independent of either group (MD, HC), time period (pre, post), or site of stimulation (left, right). Other motifs, for example, F3 *↔* Fz, are primarily present during the pre-stimulus period. Such motifs might represent processes of focusing attention to the next stimulus, preparation of the central resources, and so forth. Interestingly, there are several motifs that are specific to MD patients only, for example, C4 *↔* F4, Cz *↔* C4, or Cz *↔* Pz, while others are specific to HC subjects, for example, C3 *↔* Cz. The motifs specific to MD patients occur more often during the pre-stimulus period. They are concentrated on the central electrodes and electrodes on the right hemisphere. This might reflect the role of the right hemisphere in the processing of emotions and mood, especially in MD patients [45, 47]. In contrast, the motif specific to HC subjects is the only one that is also specific for the processing in the post-stimulus period. Therefore, it is probable that it represents the processing of the noxious stimulus itself. One might wonder that MD patients do not exhibit such a motif (or any other motif specific during the post-stimulus period); however, it should be mentioned that MD patients have been found to exhibit higher pain thresholds [42, 46], lower sensitivity to experimental nociceptive stimulation [46, 56], and/or lower processing of C-fiber nociceptive activation [57].

Motifs of size 3 (Table 2) also show differences concerning groups, time period, and stimulated site. Thus, the motif P4-Fz-F3 (motif 1, Table 2) is present in the pre- and the post-stimulus period for left and right stimulation both for MD and HC, probably representing baseline activity or brain activity that is independent from stimulation and group. Other motifs can be found only during the pre-stimulus period, for example, P4-Cz-F3 (motif 2, Table 2) or Cz-Fz-C3 (motif 4, Table 2). It is likely that these motifs are part of the network that prepares the brain for the next stimulus. Another motif occurred only before and after stimulations of the left site and before stimulation of the right site (P3-P4-Cz, motif 7, Table 2). One might speculate that it represents attentional processes before stimulation is also involved in the information processing when the left hand was stimulated. Many of these motifs involve the right parietal electrode P4 further supporting the notion of possible attentional processes. There are also motifs specifically found for stimulations of the right hand (C4-F4-Fz, motif 9 and P4-C3-Fz, motif 10, both in Table 2). All the motifs of size 3 mentioned above are independent of the group, thus representing activity for both HC and MD subjects. Interestingly, there are also motifs that differentiate between MD patients and HC subjects. Thus, the P3-P4-Fz (motif 5, Table 2) occurred in MD patients during the pre-stimulation period whereas it was found only after stimulation in HC. This might be a hint that the processing during the prestimulation time in MD involves some networks that resemble the (possibly affective) processing of noxious stimulation in HC subjects.

These results offer a number of intriguing insights into various patterns of directed interactions associated with the processing of painful and, therefore, salient stimuli, characteristic of both groups over the course of time during the experiment. However, the concept of motif detection remains controversial and questions remain. First, it is clear that motif detection misses any functional meaningful subnetworks that appear only infrequently. Conversely, subnetworks that appear with significant frequency are not necessarily important for the functioning of their network. Another criticism refers to the claim that the occurrence of specific motifs is characteristic for a certain network or a type of networks. It seems that some motifs of a network's motif distribution might occur due to contingencies in the network structure and due to topological effects known as spatial clustering (closeness of vertices in topological space or in attribute space) [31]. A test for an underlying geometric arrangement in real-world network topology has been proposed in [58]. The test is basically a comparison of invariant ratios of the numbers of certain subnetworks in geometric random network models with the same ratios obtained from the real-world network. In the same study, it has been found that the ratios in examplary real-world networks generally differed from the ratios in geometric random networks. Thus, the authors concluded that network motifs in many real-world networks are not solely captured by geometric constraints but instead arise due to additional functional optimization of network topology. Likewise, a preceding study used subnetwork significance profiles and subnetwork ratios obtained from examplary real-world networks and either geometric- or preferential-attachment networks to show that spatial clustering does not affect the number of occurrences of the majority of subnetworks and can also be ruled out as the primary mechanism that forms the structure of the real-world networks [59].

The potential ambiguity of the structure-function relationship of subnetworks and the influence of selection pressure versus variability operators on network topology, as well as the role of entanglement of subnetworks with the rest of the network, has also been debated [60]. In the context of assigning functionality to motifs, it has been argued that topological information on subnetworks must be complemented with information on parameters which describe the dynamic properties of the system, as motifs show different (and even opposing) dynamic behavior for different ranges of parameter values [61]. It has also been shown that an alleged dynamic behavior of motifs is strongly affected by the global and local dynamics of the entire network since motifs are not isolated within the network but rather are functionally interacting with many other surrounding parts of the network [61]. Investigation of the functional dependence of motifs on their context and the incorporation of parameters in the assignment of functionality to motifs is lacking in current studies; answering these analytical challenges remains a topic for further research. However, isolated network motifs have been tested experimentally for their regulatory functions as recurring circuits in bacteria and yeast transcription networks [62, 63]. The experimental studies confirmed theoretical predictions and could assign specific modes of molecular information processing to distinct motifs in these networks. Therefore, it has been shown that network motifs appear to be main building blocks of transcription networks. In principle, the role of network motifs in different systems can be examined experimentally, too.

Yet although these critiques underline potential limitations and pitfalls in assigning functionality to motifs (which is the reason for detecting them), it surely does not invalidate the concept of furthering the understanding of a network's functionality and uncovering its design principles by first analyzing local functional substructures, and then combining this information to infer network behavior at a global level. Moreover, this criticism in its entirety does not hold for our use and interpretation of network motifs, because we are primarily interested in obtaining patterns of interactions that are overrepresented in a sample of networks. It is solely this overrepresentation that allows for an interpretation of these network motifs as a characteristic of this sample. At the same time, we ignore those patterns of interactions that are not overrepresented. In this respect, our approach might be seen as a tool that simplifies the intricate topology of each member in a sample of networks by thinning out interactions that are less important for the sample of networks. Finally, after this simplification, we are able to compare different samples of networks, for example, samples of ECNs. Currently, given the outlined criticisms and lack of neurophysiological knowledge on pain processing, an understanding of the information processing roles network motifs carry out in ECNs is not yet attainable.

 We have applied network motif detection to unipolar data with a linked-ears reference. It has been shown previously that the reference might affect the results of such analyses. Specifically for coherence estimates, it is not possible to accurately predict reference effects without an accurate volume conductor model and prior knowledge of all source locations [64]. Thus, the current underlying connectivity analysis applies to the sensor space with linked ears as reference rather than to the source space. Consequently, the motif detection focuses on network motifs at the sensor level. Therefore, the current view on anatomical locations of motifs might only serve as a cautious hint with reference to anatomical sources.

 The underlying gPDC analysis has been performed on the basis of SEPs, where a multitrial estimator was applied to estimate the autoregressive model parameters [51]. For it, all raw single trials were provided separately to the estimator without any prior averaging. Thus, an explicit separation of ongoing and evoked activity [65] was not carried out. In the post-stimulus condition, the identified effective connectivity patterns, as well as the derived network motifs contain a certain amount of information associated to ongoing activity. For this reason a pre-stimulus condition was also studied in order to investigate effective interactions based solely on ongoing activity. It has been shown in [51] that the stimulus resulted in significant gPDC changes in both groups. As a consequence, we show alterations of network motif appearances associated to the stimulus.

The design of a suitable null model defines the notion of randomness and is crucial for distinguishing regular topological effects from true topological contingencies in the sample of ECNs and thus is crucial for obtaining valid results [31]. At the present time, there is no established theoretical background for choosing null models that fit to given network data and thus it is not clear which network properties might be incorporated into a good null model. The null model widely employed in motif detection preserves the degree sequence of the input network, which is a basic property on the vertex level that ultimately affects many other properties of the network. Studies that make use of this somewhat more elaborate null model rely on algorithms for generating very large sets of random networks out of the original network that all hold the desired property. These simulations are very time consuming and so is the following counting of subnetwork appearances in the obtained random ensemble. On the other hand, our analytical statistical test is computed much faster (within seconds implemented in the MATLAB programming language) than simulating random networks and counting their subnetwork occurrences, but at the cost of simpler assumptions being made for the null model which accounts for the mean number of edges of the input network sample.

## 4. Conclusions

 We have presented an approach to analytically detect network motifs in a sample of directed networks with pairwise different vertex labels. The importance of choosing an appropriate null model random network that contrasts topological regularities of the input networks with topological contingency is outlined. Clearly, a refinement of our analytical null model, which accounts for the average number of edges in a sample of networks, is desirable. Such a refinement is currently under investigation. Nonetheless, we have demonstrated that our approach to network motif detection is suitable to act as a filter to reveal locatable patterns of directed interactions that might be interpreted as characteristic for each of several group-specific samples of ECNs. These networks originate from effective connectivity data obtained in our previous study that investigated cortical activity before and after painful stimulation of patients with major depression and healthy control subjects [51]. The detected motifs on the one hand yield a compact description of recurring important topological elements in a sample of ECNs. On the other hand, they allow for a comparison of different samples of ECNs, which was as yet not attainable. The sample-specific network motifs of ECNs can now be investigated in more depth to gain further understanding of neurophysiological processes in both groups during the anticipation and processing of painful stimuli. This in turn should contribute to a deeper understanding of the relationship between pain and depression.

## Figures and Tables

**Table 1:** The mean number of all 2-motifs in the eight samples of effective connectivity networks (ECNs). The 2-motifs represent important interactions before and during the processing of painful electrical stimuli. The samples originate from all combinations of the group assignment. MD—patients suffering from major depression, HC—healthy control subjects, left and right—stimulated side, pre and post—time window with respect to the stimulus condition.

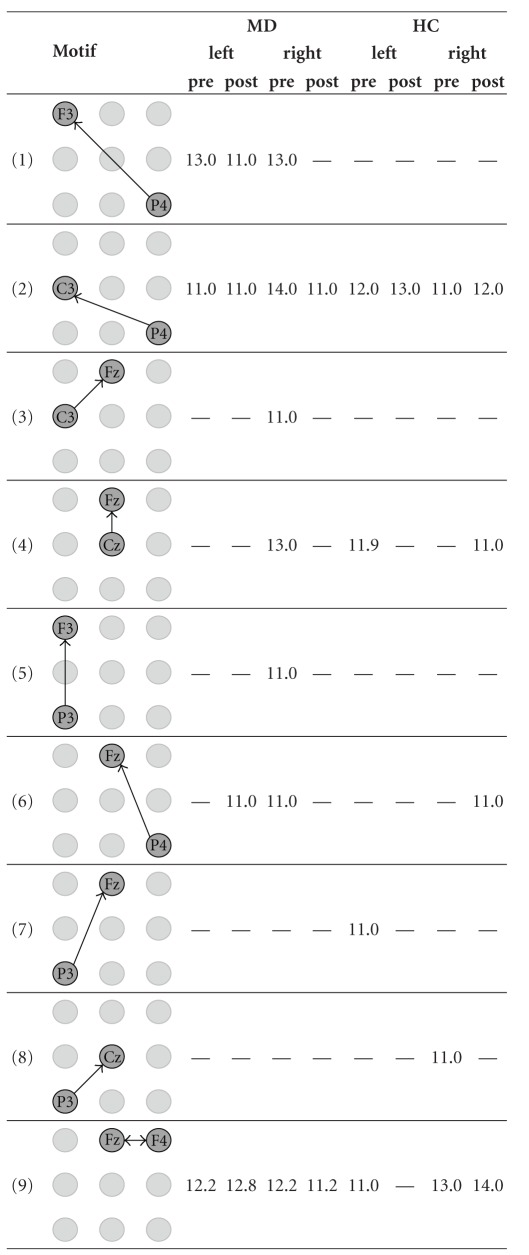 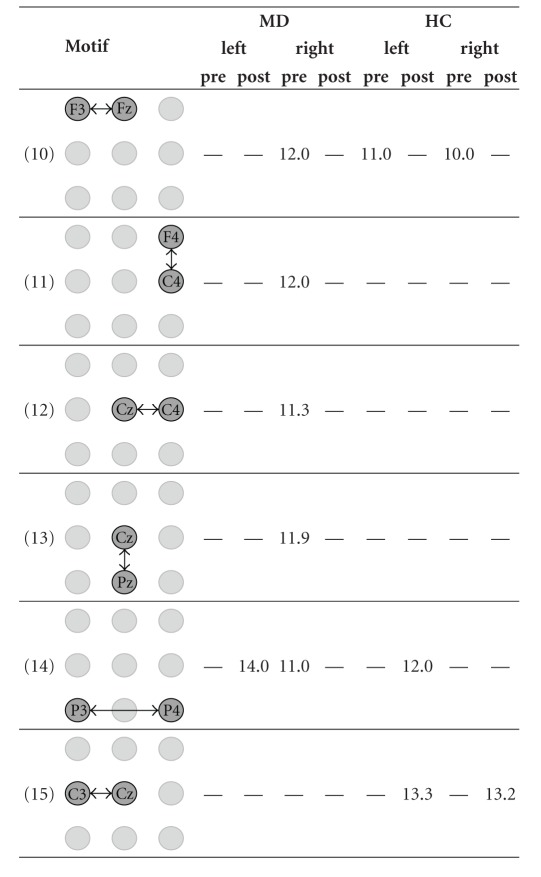

**Table 2:** The mean number of 3-motifs with interesting similarities and differences in the eight samples of effective connectivity networks (ECNs). The 3-motifs represent important patterns of interactions before and during the processing of painful electrical stimuli. The samples originate from all combinations of the group assignment. MD—patients suffering from major depression, HC—healthy control subjects, left and right—stimulated side, pre and post—time window with respect to the stimulus condition.